# Supplementary material for: Effects of entrepreneurial orientation on social media adoption and SME performance: The moderating role of innovation capabilities
Source: PLoS One. 2021 Apr 28;16(4):e0247320. doi: 10.1371/journal.pone.0247320 (PMC8081172; doi:10.1371/journal.pone.0247320)
Supplement: S1 File — (DOCX) [file pone.0247320.s001.docx]

**Questionnaire**

**Instructions**

1. Use a tick mark √ to show your preference in the box provided
2. No need of writing any name, mobile, number or address.

**Part 1: Demographics Information**

*Gender* **Male Female**

*Age (Years)*

**Less than 26 26 – 35 36 – 45 Over 45**

*Education:*

**Secondary or Basic Undergraduate Master Other**

*Position*

**Owner Executive Manager**

*No. of Employees*

**Less than 9 11 – 50 51 – 250**

*Industry Sector (mention the sector in box)*

*Firms level of Utilization of Social Media*

**Minimal Basic Moderate Extensive**

*Use of SM as a Marketing Tool*

**Very Little Little Quite A lot Extensive**

*Budget allocated (%)*

**Less than 25 26 – 50 Greater than 50**

*Firms Location*

**Punjab Sindh KPK Baluchistan**

*Social Media Platform Used*

**Facebook Twitter Instagram WhatsApp**

**LinkedIn YouTube Google+ Other**

**PART 2: Basic information -** Please indicate your opinion by marking the appropriate box on the five point Likert scale where (***1 = Strongly Disagree, 2 = Disagree, 3= Neutral, 4 = Agree, 5 = Strongly Agree***)

| **S.N** | ***Entrepreneurial Orientation*** | | **1** | **2** | **3** | **4** | | **5** |
| --- | --- | --- | --- | --- | --- | --- | --- | --- |
| 1 | “Innovations are appreciated above everything else” | |  |  |  |  | |  |
| 2 | “We emphasize R&D, technological leadership and innovativeness instead of trusting only those products and services, which we have traditionally found to be good” | |  |  |  |  | |  |
| 3 | “We emphasize risk taking” | |  |  |  |  | |  |
| 4 | “In our company, many people want to take risk” | |  |  |  |  | |  |
| 5 | “Within the last five years, we have brought several new products or services to the market” | |  |  |  |  | |  |
| 6 | “We intend to get into markets before our competition” | |  |  |  |  | |  |
| 7 | “We are typically ahead of competitors in presenting new products or procedure” | |  |  |  |  | |  |
|  | “In our company people want to be first in the markets” | |  |  |  |  | |  |
|  | ***Social Media for Marketing*** | | **1** | **2** | **3** | **4** | **5** | |
| 1 | “It helps to conduct marketing research” | |  |  |  |  |  | |
| 2 | “It helps to get referrals (word of mouth via likes, shares and followers in Facebook)” | |  |  |  |  |  | |
| 3 | “It helps to advertise and promote product/services” | |  |  |  |  |  | |
| 4 | “It provides aids to deliver customer services” | |  |  |  |  |  | |
|  | ***Customer Relationship*** | | **1** | **2** | **3** | **4** | **5** | |
| 1 | “It helps to develop customer relations” | |  |  |  |  |  | |
| 2 | “Communicate with customers” | |  |  |  |  |  | |
| 3 | Conduct customer service activities” | |  |  |  |  |  | |
| 4 | “Receive customer feedback on existing product/services | |  |  |  |  |  | |
| 5 | “Receive customer feedback on new/future product/services” | |  |  |  |  |  | |
| 6 | “Reach new customers” | |  |  |  |  |  | |
|  | ***Information Accessibility*** | | **1** | **2** | **3** | **4** | **5** | |
| 1 | “It helps to search for general information” | |  |  |  |  |  | |
| 2 | “Search for competitor information” | |  |  |  |  |  | |
| 3 | “Search for customer information” | |  |  |  |  |  | |
|  | | ***Innovation Capabilities*** | **1** | **2** | **3** | **4** | **5** | |
| 1 | | “There is constant generation of new product or service ideas in this firm” |  |  |  |  |  | |
| 2 | | “We are constantly having R&D funds in order to search for new ways of doing things” |  |  |  |  |  | |
| 3 | | “There is creativity in our methods of operation” |  |  |  |  |  | |
| 4 | | “This firm is usually a pioneer in the market” |  |  |  |  |  | |
| 5 | | “This firm is able to introduce new product or services every five years due to continuous support provided to R&D” |  |  |  |  |  | |
|  | | ***Performance*** | **1** | **2** | **3** | **4** | **5** | |
| 1 | | “Improved customer relationship” |  |  |  |  |  | |
| 2 | | “Service quality” |  |  |  |  |  | |
| 3 | | “Customer engagement” |  |  |  |  |  | |
| 4 | | “Increase in company/brand visibility and reputation” |  |  |  |  |  | |
| 5 | | “Increased customer loyalty and retention” |  |  |  |  |  | |
| 6 | | “Enhance the customer service” |  |  |  |  |  | |
| 7 | | “Increase product/ service awareness among customers and increase market share” |  |  |  |  |  | |
