# Supplementary material for: Effects of entrepreneurial orientation on social media adoption and SME performance: The moderating role of innovation capabilities
Source: PLoS One. 2021 Apr 28;16(4):e0247320. doi: 10.1371/journal.pone.0247320 (PMC8081172; doi:10.1371/journal.pone.0247320)
Supplement: Attachment — Submitted filename: recomendations.DOCX [file pone.0247320.s003.DOCX]

Reviewer report for Plos One jounal

Manuscript ID: PONE-D-20-22933

Manuscript Title: Effects of entrepreneurial orientation on social media adoption and SME performance:
the moderating role of innovation capabilities

Comments

1- I do believe that theoretical contribution remains silent, further, the practical contribution was not articulated well, in turn, make the contribution part unconvincing. The authors should build the contribution part again and mention it in the introduction section.
2- The motivations are not clear. In other words, the manuscript does not show the following (1) Why is this study important? (2) Why conducting this topic in a developing setting “Pakistan” may create a new contribution? Means what are the differences that make significant insights (3) Prior studies almost have investigated the relationship between EO and performance as authors mentioned, but the authors did not explicitly report what the new measures used in the study to show the new contribution in this study.
3- The theoretical part is weak, I suggest authors to do more effort in this context.
4- Although I am not that much familiar with survey research but I find the results discussion section is very weak.
5- I have concerns regarding the SMEs performance measurement. Which means authors built the sub-questions unclearly. For example. FP7 “Increase awareness and market share”

Increase awareness whom? and awareness of what? Also I did not find the main question of each group. i.e., what is the main question of the dependent variable (SMEs performance)?

6- Hypothesis 6 suggests that innovation capabilities moderates the relationship between SM and SMEs performance. The point is to there would be better if there is a question about R&D expenses to know the moderating effect of IC. One more thing, there is a question regarding budget allocation. So, I suggest the authors check the moderating role of the budget allocation on the relation between SM and SMEs performance. As an alternative or additional measure for IC. And see what is the result.

7- The manuscript has some incomplete ideas for example

(1) “**Grounded on resource-based view (RBV) theory, EO is considered a key element for organizations competing in a digital environment (Parveen et al. 2016).”** Why EO is a key element for OC?

(2) Paragraph 6 in the introduction part ( because no pages’ number) ….**However, there is no consensus on the mediation effects** (of what ??) , **with limited research on the casual mechanism of how/why EO impacts other constructs** (such as what).
