# Supplementary material for: Effects of entrepreneurial orientation on social media adoption and SME performance: The moderating role of innovation capabilities
Source: PLoS One. 2021 Apr 28;16(4):e0247320. doi: 10.1371/journal.pone.0247320 (PMC8081172; doi:10.1371/journal.pone.0247320)
Supplement: Attachment — Submitted filename: Reviewers response(1)(1).docx [file pone.0247320.s004.docx]

**Reviewer’s response form**

| **Reviewer Comments** | **Response** |
| --- | --- |
| 1. I do believe that theoretical contribution remains silent, further, the practical contribution was not articulated well, in turn, make the contribution part unconvincing. The authors should build the contribution part again and mention it in the introduction section. | Thank you for this valuable feedback. We have updated as per suggestion  Added and explained theoretical contribution in terms of our study and also added practical contribution of our study from line number 53-59 and from line numbers 517-596. |
| 2. The motivations are not clear. In other words, the manuscript does not show the following (1) Why is this study important? (2) Why conducting this topic in a developing setting “Pakistan” may create a new contribution? Means what are the differences that make significant insights (3) Prior studies almost have investigated the relationship between EO and performance as authors mentioned, but the authors did not explicitly report what the new measures used in the study to show the new contribution in this study. | Thank you for this valuable feedback. We have added a short paragraph of 150 words from line numbers 106-117. in which we have clearly stated the motivation and why we opt to study this topic |
| 3. The theoretical part is weak, I suggest authors to do more effort in this context. | Thank you for this valuable feedback. We have updated theoretical part on line numbers 53-59, then from 63-69, also at lines 102 and 103 and then at lines 106-117 to make it strong and justifiable. |
| 4. Although I am not that much familiar with survey research but I find the results discussion section is very weak. | Thank you for this valuable feedback. We have explained the results in terms of practical implications and also in terms of subjective measure. Furthers, we have re-written the discussion section and explained things in more details. |
| 5. I have concerns regarding the SMEs performance measurement. Which means authors built the sub-questions unclearly. For example. FP7 “Increase awareness and market share”  Increase awareness whom? and awareness of what? Also I did not find the main question of each group. i.e., what is the main question of the dependent variable (SMEs performance)? | Thank you for this valuable feedback. We have cleared this point in the table 4.  Increase product/ service awareness among customers and increase market share” |
| 6. Hypothesis 6 suggests that innovation capabilities moderates the relationship between SM and SMEs performance. The point is to there would be better if there is a question about R&D expenses to know the moderating effect of IC. One more thing, there is a question regarding budget allocation. So, I suggest the authors check the moderating role of the budget allocation on the relation between SM and SMEs performance. As an alternative or additional measure for IC. And see what is the result. | Thank you for this valuable feedback. we have cleared this point a little because in our research we have asked such questions which directly measure the R&D in the organization in table 4.  We are constantly having R&D funds in order to search for new ways of doing things  This firm is able to introduce new product or services every five years due to support provided to R&D.  We have checked the proposed moderating role of budget allocation but unfortunately could not find relevant literature to propose construct and valuable results by running moderating effect of budget allocation. |
| 7. The manuscript has some incomplete ideas for example  (1) “**Grounded on resource-based view (RBV) theory, EO is considered a key element for organizations competing in a digital environment (Parveen et al. 2016).”** Why EO is a key element for OC?  (2) Paragraph 6 in the introduction part ( because no pages’ number) ….**wever, there is no consensus on the mediation effects** (of what ??) , **with limited research Hoon the casual mechanism of how/why EO impacts other constructs** (such as what). | Thank you for this valuable feedback. we have explained that why EO is considered a key element for the organizations on page no 2 on line numbers 53-59.  Thank you for your feedback, we have cleared this statement on line numbers 102-103. |
